# The evolution of online ideological communities

**DOI:** 10.1371/journal.pone.0216932

**Published:** 2019-05-22

**Authors:** Brittany I. Davidson, Simon L. Jones, Adam N. Joinson, Joanne Hinds

**Affiliations:** 1 School of Management, University of Bath, Bath, Avon, United Kingdom; 2 Department of Computer Science, University of Bath, Bath, Avon, United Kingdom; University of Texas at Austin, UNITED STATES

## Abstract

Online communities are virtual spaces for users to share interests, support others, and to exchange knowledge and information. Understanding user behavior is valuable to organizations and has applications from marketing to security, for instance, identifying leaders within a community or predicting future behavior. In the present research, we seek to understand the various roles that users adopt in online communities–for instance, who leads the conversation? Who are the supporters? We examine user role changes over time and the pathways that users follow. This allows us to explore the differences between users who progress to leadership positions and users who fail to develop influence. We also reflect on how user role proportions impact the overall health of the community. Here, we examine two online ideological communities, RevLeft and Islamic Awakening (N = 1631; N = 849), and provide a novel approach to identify various types of users. Finally, we study user role trajectories over time and identify community “leaders” from meta-data alone. Study One examined both communities using K-MEANS cluster analysis of behavioral meta-data, which revealed seven user roles. We then mapped these roles against Preece and Schneiderman’s (2009) Reader-to-Leader Framework (RtLF). Both communities aligned with the RtLF, where most users were “contributors”, many were “collaborators”, and few were “leaders”. Study Two looked at one community over a two-year period and found that, despite a high churn rate of users, roles were stable over time. We built a model of user role transitions over the two years. This can be used to predict user role changes in the future, which will have implications for community managers and security focused contexts (e.g., analyzing behavioral meta-data from forums and websites known to be associated with illicit activity).

## Introduction

Online communities provide users with rich sources of information, the ability to exchange ideas, expertise, and the opportunity to form social connections [1]. This can be a source for good, for instance, research has revealed the positive effects of online communities for support [2–5]. However, this is not always the case. There are malevolent online communities, for example, (specific areas of) 4chan, also described as the “*internet hate machine*” [6]. Other examples include the recent “involuntary celibate”, or “incel” movement, found in subsections of 4chan, Reddit, other websites (that may have had links to the Canadian terror attack in 2018 [7,8]), or specific ideologically motivated forums with malicious intent and the aim to radicalize members [9].

Users in online communities are largely transient in nature, which tends to cause a high churn rate within these communities [2,11] and complicates the understanding of user behavior due to a lack of a consistent user-base. However, despite high churn rates, there tends to be a core set of members that continue to contribute, share, and retain knowledge within a community [10], which is important for new joiners. While most users will inevitably leave the community, there is often an influx of new users that serves to refresh and maintain membership levels.

Despite this, relatively little is known about how (some) users evolve into valued members once they have become engaged within online communities. Previous research has addressed specific stages in the lifecycle of a community member. For instance, how users join and become accepted [12–14], or how language is used by those in leadership positions [15]. Various researchers have also examined the roles that a user may adopt in a community [15–17], however this work tends to treat a role as a pattern of behavior that is static (rather than dynamic), such that once a role is established, it rarely changes. This is perhaps a limiting assumption, in light of research that has found that behavioral patterns and roles change over time, both on- and offline [16,19,20]. We adopt various roles throughout our lifetime–changing subtly and substantially according to the context and those around us at the time [19].

In the present research, we analyze the roles that users adopt within two moderately-sized online communities; one political discussion forum, RevLeft (denoted as community A) (N >1000 at each six-month time slice over a period of two years) and one religious discussion forum, Islamic Awakening, denoted as community B (N = 849). Since data collection, both online communities have closed. Community A was a far-left forum with many groups and threads relating to anarchy and various forms of communism [20]. Community B described itself as a “*small effort and a humble contribution […] towards the global revival of Islam*” [21].

In the first instance (Study One), these roles are treated as stable across the tenure of the community members in order to build a classifier. This classifier is then used to categorize members of one community in unique six-month time slices (Study Two), which allows us to study any movement between roles across time. We also consider the stability in user numbers per role, as an indicator of community heath, as suggested in the work of Angeletou et al. [22].

We also analyze user churn in community A. Rate of churn is a long-standing concern for community managers who attempt to retain users [1,23,24]. We analyze all role transitions over the two-year period (N = 7,712) in order to understand the distribution of users’ changing roles, remaining in the same role, or potentially leaving the community.

We ground our work within a framework conceptualizing user engagement and leadership: the Reader-to-Leader Framework (RtLF) [25], which describes how users transition from being passive “readers” to potentially active community “leaders”. Hence, the RtLF provides us with a valuable theoretical lens through which we examine user behavior within online communities, focusing on user role changes over time. By grounding our analyses within this framework, we discuss how this is important for those moderating and managing online communities. Finally, we consider the role transition pathways that users make over time and provide a method and theoretical underpinning to understand these various pathways.

### Theoretical background

#### Conceptualizations of user engagement

**Fig 1:**
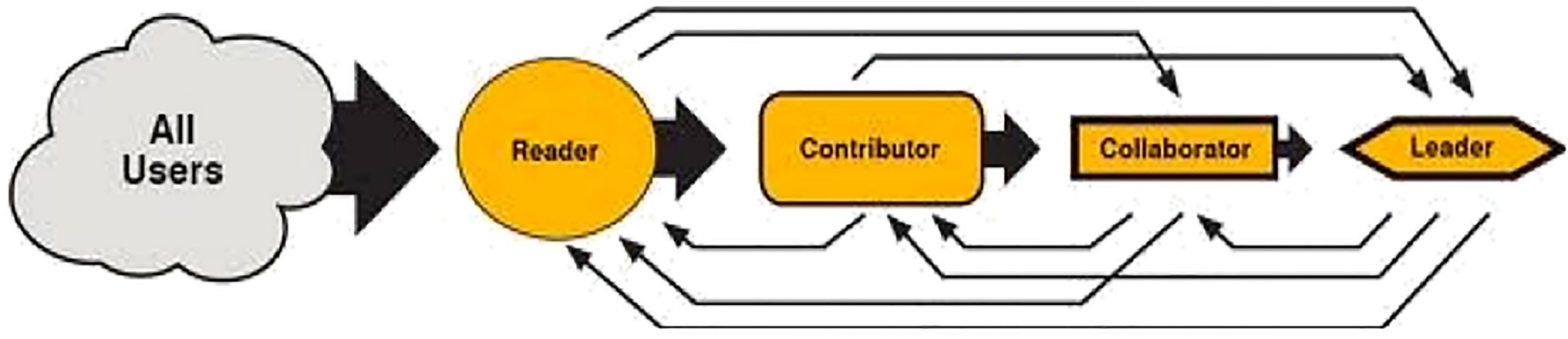
Reader-to-Leader Framework (RtLF).

Engaged, active users are the lifeblood of an online community. It should be of little surprise that there have been numerous attempts to study not only user engagement, but also the ways in which users move from passive consumers to active creators within a community. One relatively well established approach is the Reader-to-Leader Framework (RtLF) [25] (Fig 1). The RtL framework describes four roles that users can adopt in online communities:

- **Reader**–visiting, reading, searching, returning
- **Contributor**–posting, reviewing, rating
- **Collaborator**–engaging with other members, collaborating to create content
- **Leader**–mentoring new joiners, setting policies and monitoring users, promoting participation

Preece and Schneiderman [25] state that although these categorizations are not exhaustive, they describe the participation of many users. While the RtLF does not specify quantities of users at each stage in the framework, they explicitly state that the proportion of users moving towards a leadership significantly diminishes.

This inequality in participation has been noted before–for instance, Nielsen [26] described the 90-9-1 rule of online participation, stating that 90% of users online will lurk–where they “*read or observe*, *but do not contribute*” [26], 9% will have small contributions over time, and the final 1% drive the majority of conversation. Both of these conceptualizations attempt to describe the wide variance of online user participation. The RtLF has been extensively used since its initial publication in 2009 and remains influential when examining online user behavior [27,28]. While it does describe how users can progress from being a reader of content, to a contributor and collaborator sharing their own content, to potentially a leader guiding narratives within the community, it lacks clear criteria for what behaviors constitute each role, and how transitions actually happen. The framework suggests most users follow a linear progression through the successive levels, with a decreasing proportion of users moving from one role to the next (illustrated by the size of arrows in Fig 1). However, it also suggests that users can move in a non-linear fashion [29]. For example, a portion of users might be able to make the direct transition to a position of leadership, having previously contributed very little to the community. Moreover, the RtLF framework does not offer a strong indication of the proportion of users that make such transitions, nor does it shed light on the characteristics of users that follow particular pathways of participation. The present study addresses these issues by examining users over time with reference to the RtLF in order to quantify proportions of users who progress in both linear and non-linear pathways. While it is useful to understand general patterns in users’ paths through their usage lifecycle, we contend that it is also useful to understand the specific roles and trajectories of certain individuals. We propose that understanding user roles will aid understanding of subtle differences in groups of users, which will then offer insight into the dynamic within the community. Such insights could enable us to understand what makes a user maintain, increase, decrease, withdraw participation, and recognize factors that differentiate users that follow different paths through the various levels. Forecasting the future actions of users given their past and present trajectories is likely to be useful for analyzing the health of online communities, and for enabling designers and managers to identify characters such as “rising stars” or “fading leaders” at an early stage.

#### Understanding changes in engagement

An extensive body of literature examines the different roles that people assume within online communities [16,27,28,30]. In particular, *social role theory* considers behavior to be the enactment of socially defined categories (e.g., teacher, student, manager) [18]. A social role consists of norms, expectations, and behaviors that a person tends to fulfill. Gleave et al. [15] define social roles as, “*a combination of social psychological*, *social structural*, *and behavioral attributes*”. Social role theory implies that in order to change behavior (e.g., increased participation) it would be necessary to change roles. Therefore, one might hypothesize that role changes act as an indicator of transitions within the RtLF.

It has been argued that each user possesses a set of beliefs, which may or may not align with the community or group beliefs online or in offline groups. However, as users integrate and interact with a community, they will enter a process of adopting, adapting, and potentially discarding prior beliefs of roles. Hence, every community (on- or offline) is unique and varies in terms of members, behavior, and communications [31,32]. Schmader and Sedikides [33] proposed a conceptual framework, State Authenticity as Fit to the Environment (SAFE), which provides an additional way to understand engagement. In SAFE, if the individual has a good self-concept, goal, and social fit to the environment, they are more likely to approach and engage.

A number of different roles have been identified in studies of online discussion groups (see Table 1). These roles have primarily been identified through ethnographic study of the content of interactions [34,35], although some effort has been made to use behavioral metrics to recognize these roles [36,37]. The roles that emerged from these studies have shown various levels of depth in terms of a) how specific a role is, b) how dynamic a role is, and c) how dynamic the network is. Furthermore, changes in roles are often dismissed in the analysis.

**Table 1:** Examples of roles identified in previous research.

Author(s)	Roles Identified
Golder & Donath [38]	Newbie, Celebrity, Lurker, Flamer, Troll, Ranter
Turner, Smith, Fisher, & Welser [37]	Answer person, Questioner, Troll, Spammer, Binary poster, Flame warrior, Conversationalist
Campbell, Fletcher, and Greenhill [39]	Big man, Sorcerer, Trickster
Chan, Hayes & Daly [32]	Joining conversationalist, Grunt, Taciturn, Popular participants, Popular initiator, Supporter, Ignored
Pfeil, Svangstu, Ang, & Zaphiris [40]	Moderating supporter, Central supporter, Active member, Passive member, Technical expert, Visitor
Welser, Lin, Cosley, Dokshin, Smith, Kossinets, & Gay [16]	Substantive experts, Technical editors, Counter vandalism contributors, Social networkers
Panteli [28]	Emergent leaders, Appointed leaders, Community founder, Sustaining leaders
Arazy, Lifshitz-Assaf, Nov, Daxenberger, Balestra, & Coye [27]	Role-Article Samplers, Role Embracing, Article Embracing, Role-Article Polymathing

Both Gleave et al. [15] and Welser et al. [16] visualized these systematic patterns of behavior as forms of “structural signatures”, which provide insight into the overall role distributions within a community. Further, the topologies of communities rely on user individuality in terms of their behavior and levels of participation in order to group them into roles. This then could be used to provide insight into the health of an online community [22]. Our work aims to improve the classification of community members by considering various behavioral metrics (see Data Metric Development section). Chan et al. [32] used nine features to profile users, including popularity, reciprocity, length of interaction, initiation, neighbor’s roles, and volume of communication measures, which is a similar set of features utilized in the present studies. Arazy et al. [27] examined role-transitions within Wikipedia specifically, where they categorized users into four-types of role changes, which sheds light on the fact that user behavior is indeed active and dynamic. Similarly, Campbell, Fletcher, and Greenhill [39] explore users changing roles within communities, however, they focus more specifically on conflict between roles within communities.

We extend this work by finding social roles in two online communities (Study One: community A and B) and by examining these roles over time (Study Two: community A). First, we analyze the user role changes over time to investigate the most common role transitions for users, for example, how often do users become leaders, and do those leaders often fall from grace? Then, by analyzing more specific role transitions, we are able to understand the proportions of users who engage in various role transitions. This analysis reveals what transitions are more common, which could act as a basis to predict how a user may transition in future.

#### Community health

The relationship between the health of an online community and user churn has two competing schools of thought. The first, and perhaps more traditional perspective, attempts to understand how community managers can increase user engagement and retain as many users as possible, as high churn rates are deemed as negative [1,23,41]. However, one might argue that user churn is a natural occurrence of online communities and one should rely more on whether the community is growing overall in size, as opposed to focusing on users becoming inactive only. The second approach argues that the high churn rate is actually a positive trait for an online community. For instance, Soroka and Rafaeli [24] suggest that if all users (including readers or lurkers) were actively engaging in the community, this could dilute the knowledge and create unnecessary “*noise*” (e.g., off-topic discussion or too much content), which can be destructive. Further, if all users were constantly posting–who is listening? Hence, a high number of reading users (or “lurkers”) is not necessarily a negative trait in an online community [22,42]. Further, Angeletou et al. [22] suggest that higher numbers of elite or popular users are a sign of a healthy community, alongside other indicators such as: having a stable distribution of user roles over time; having a mixture of roles within a community; and lower levels of “ignored” and “low engagement” users. However, Chan, Hayes, and Daly’s [32] findings demonstrate a variety of different role compositions in various online communities. Therefore, factors in community health perhaps stretch beyond role composition. Further, other work has shown that social purity (e.g., sharing the same political view) is important within social networks [43], which suggests that community health is indeed multi-faceted and unique to each community.

The present research will investigate various ways to analyze community health by considering user churn over time, number of roles found, and the stability of them over time.

Our research questions are as follows:

1. What specific roles can be identified using meta-data from online communities?
2. Do user roles change over time? What are the most common pathways and transitions for users?
3. What can we learn about the health of the community based on user roles?

The present research contains two studies. Study One utilizes the meta-data from users within communities A and B, where we used a clustering algorithm (K-MEANS) in order to detect groups of similar users. After analyzing and naming each cluster, we then map the roles established to the RtLF [25] in order to conceptualize differing levels of engagement, hierarchy, and leadership within the communities. Study Two is a time-series analysis of community A over a two-year period. Here, we are able to understand whether a user changes their behavior to the extent that their new behavioral pattern exemplifies a different role. Further, we examine the distribution of clusters over time with reference to the RtLF, which provides a theoretical grounding of the types of users that participate in communities. We also analyzed every user role change over the two-year period (N = 7,712) and used this to build a model of role transitions.

## Statement of ethics

The present study was reviewed and approved by the Ethical Implications of Research Activity (EIRA) process within the University of Bath, School of Management (reference number: 2393). IRB approval was not required for this work as it only utilized secondary data analysis.

## Data preprocessing

### Data collection

Content and meta-data were collected from two publicly accessible communities denoted: community A (RevLeft) and community B (Islamic Awakening). This data was collected via “screen scraping”. This is a technique that is similar to automated cut-and-pasting from online webpages. This was done by a custom PERL/MySQL tool, which collected data securely from the Internet utilizing a Privoxy/TOR chain. Where needed for forum access, cookies were supplied with HTTP requests made by the tool and were regularly rotated to ensure anonymity. Data errors were captured by validating the number of fields of each type that had been identified and extracted by the HTML parser, and regular expressions from each webpage. All validation errors, each URL scraped, and cookie and IP rotations have been logged and retained in order to monitor scraping accuracy. No scraping behind logins was conducted and only publicly available data was collected and stored in a MySQL database, which complies with the Terms of Service at the time of data collection. User ID’s were encrypted via the MD5 hash algorithm to ensure user identity and privacy.

When these forums were scraped, community A had approximately 1.49 million posts and 11,778 active members. Community B had approximately 500,000 posts and 3,205 active members. Both communities A and B have since been closed.

The two present studies only use six months (Study One–A and B) and two years (Study Two–A only) worth of these data archives. This was due to the early years of the archive capturing the start of the forums, therefore, we focused only on the most recent data (at the point of data collection) to analyze as this is when the forums were fully-established. For Study One, we used the most recent six-months’ worth of data compiled into one data frame. Study Two used two years’ worth of data, which was split into four six-month time slices, to allow for a time-series analysis. While having six-month time slices is a broad window within which to classify users into a single behavior-based role, we selected this time period in order to avoid capturing minor or temporary fluctuations in behavior, as opposed to sustained changes in behavior as reflected as changes in roles.

### Data metric development

From all scraped data, we derived several types of behavior metrics (shown in Table 2). As we intended to group similar users into clusters, we needed to develop metrics in order to compare users against each emerging role, and the community [22]. Typically, when examining user online meta-data, prior work had emphasized a variety of features that should be included. For example, Chan, Hayes, and Daly [32] created structural features (providing an indication of communication between unique users), reciprocity features (how much users reply to one another), popularity features (number of in-neighbors e.g., those who replied to that user, or thanks rate), persistence features (e.g., length of conversations and number of threads or subforums in which users post), and initialization features (how often a user starts a thread). Angeletou et al. [22] also utilized similar metrics overall, which aligns with the features employed in our work. Hence, prior work has demonstrated the importance of the metrics used in order to distinguish between roles that users adopt in online communities.

**Table 2:** Metrics used in cluster analysis for communities A and B.

**Structural Features**	**In-Degree**	Total number of unique network neighbors replying to (or quoting) a user
	**Out-Degree**	Total number of unique network neighbors receiving posts from (or being quoted by) a user
**Content Features**	**Word Count**	Mean average word count for all of a user’s posts
	**Percentages**	Percentage of a user’s posts that contain question marks (excluding within URLs)
	**Percentage URLs**	Percentage of a user’s posts that contain URLs
**Popularity Features**	**Thanks Rate**	Mean average number of thanks per post. Calculated as: Total Number of Thanks Received / Total Number of Posts Made
**Initiation Features**	**Initiation Ratio**	Calculated as: Number of threads initiated/Number of threads participated in
**Diversity Features**	**No. Threads**	Total number of threads participated in
	**No. Sub Forums**	Total number of sub-forums participated in
**Persistence Features**	**Posts Per Sub Forum**	Calculated as: Total number of posts/Number of sub forums participated in
	**Posts Per Thread**	Calculated as: Total Number of posts/number of threads participated in
**Reciprocity Features**	**Percentage Bi-directional Neighbors**	Calculated as: Number of neighbors that a user has both received posts from and posted replies to/Total number of unique network neighbors

We also wanted to understand as much as possible about the content posted by users, from a meta-data perspective, hence we added additional “Content Features”, which consist of average word count, number of URLs present in posts, and the number of questions asked per post. We anticipated that this would help to distinguish between various low engagement users (or contributors referring to the RtLF). For example, Golder and Donath [38] identified “newbies”, who tend to have little knowledge but would ask many questions. Therefore, users with a high percentage of questions asked and relatively low numbers of posts might fall into this category. Similarly, we wanted to capture the number of URLs being provided in posts, as we anticipate that this could reflect users signposting information, which might be similar to Welser, Gleave, Fisher, and Smith’s “Answer Person” [44]. We also included “Diversity” features, which aim to capture the extent to which a user posts in the same threads or subforums. We believe this could be insightful, particularly for distinguishing between users who focus on a limited number of specific threads, and those who engage in a number of conversations across the broader community.

## Study One: Cluster analysis

This study utilizes all user activity during the six-month period prior to data collection. Our aim is to understand sets of similar users within both forums, based on the important behavioral features of each role. This will reveal which roles tend to lead and influence the community, who might support the leaders, and who simply follows. These roles will be mapped against Preece and Schneiderman’s [25] RtLF based on key features of each role, reputation scores (“*the opinions of all your forum users*”, based on “*how [their] posts are scored by other forum participants*” [45], which is a native, inbuilt metric), and the number of users within each role.

### Methods & results

#### Metrics utilized for analysis

We performed a K-MEANS cluster analysis using Weka 3.8.2. We used this method as it is widely used for behavioral analytics, data mining, and data science more generally [6,27,32,46,47]. It also provides easily understandable and scalable outputs [47]. However, we note that K-MEANS clustering algorithms, while widely applicable, can be sensitive depending on initial seeding [48], therefore additional attention to cluster centers and the variable input is critical.

Table 2 shows the metrics that were used during the clustering process. We also collected each user’s “Reputation” score, which was removed from the cluster analysis. Instead, we used this as an additional metric to map the outputs from the cluster analysis to the RtLF, since we expect reputation scores to increase as users progress through the RtLF.

### Ideal number of clusters

**Fig 2:**
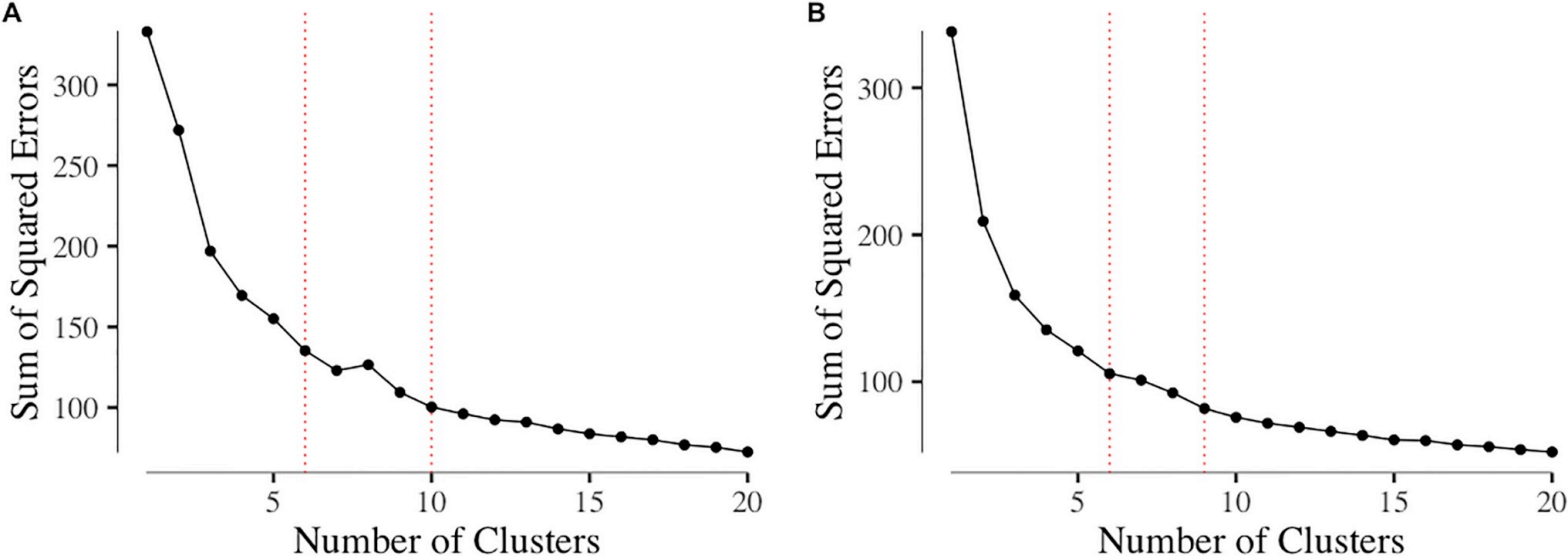
Elbow plot or “Sum of Squared Errors” plot for communities A (A) and B (B). The dotted red lines denote the upper and lower boundaries for the ideal number of clusters, *k*.

Cluster analyses are considered a form of unsupervised learning due to a lack of a defined set of classes prior to learning [49]. K-MEANS is a partitional clustering algorithm that divides the data into smaller sections called “clusters” [50]. When running the K-MEANS algorithm a pre-defined number of clusters, *k*, is required. The ideal *k* value can be found via trial and error and is highly subjective [51]. We found *k* via the Elbow Method, see Fig 2. This aims to visualize and explain the “*percentage of variance explained as a function of the number of clusters*” [50]. This means that the first few clusters will have large decreases in variance as each additional cluster continues to add information to the model. This will eventually plateau as the model does not continue to improve substantially [50]. We highlighted the boundaries of the potential number of *k* in Fig 2.

Fig 2 shows the Elbow Plots for communities A and B. Based on the elbow plot, we proceeded with seven clusters for both communities (using the same *k* value for each community as a basis for comparing how the overall composition of roles differs between communities). We note that in Fig 2(A), at *k* = 8, the elbow began to rise, showing that *k* = 7 for this community is optimal. Similarly, in Fig 2(B), the elbow begins to plateau from *k* = 7, which indicates this would be an appropriate number of clusters. Hamerly and Elkan [51] stress the difficulty of identifying *k*, where they state this tends to rely on prior knowledge. From our theoretical underpinning of the RtLF, we could only take forward *k*≥3 (reflecting the contributor, collaborator, leader distinction), however, this would be too coarse for revealing more subtle roles within each of these levels, which have been identified in previous work. In the literature referenced in Table 1, the number of roles identified varies from 3 to 8. We wanted to capture the more intricate and subsets of roles within the RtLF, hence, we proceeded with *k* = 7 as this seemed most appropriate based on Fig 2, previous literature [32,37,52], and the RtLF.

### Clusters & visualization

In order to map our clusters to the RtLF, we first need to understand what each cluster means. From looking at the centroids (or multi-dimensional mean) of each variable within each cluster, we were able to deduce how clusters differ from one another. For community A, Table 3 describes each cluster and Table 4 provides additional information regarding cluster centers. Similarly, Tables 5 and 6 relate to community B. While we use the same names for the eight roles found in both communities, there are some subtle differences. For example, the Popular users in community B had the highest overall *thanks rate*, whereas the Elite users had the highest *thanks rate* in community A.

**Table 3:** Cluster descriptions for community A. See Table 4 for more detailed information on cluster centers.

Cluster/Role Name	Description
**Newbie**	High initiation rate, highest overall number of questions asked in the community, and typically long word counts in their posts. Lowest in- and out-degree and number of posts
**Popular Supporter**	High overall metrics, particularly in- and out- degree, bi-directional neighbor degree, and thanks rate. Users were similar to the Elite users, however, overall lower in each metric
**Taciturn**	Low in all metrics, largely not engaged, as reflected by their low activity (e.g., low number of posts and connectivity)
**Conversationalist**	High number of posts per thread and initiation rates, with high bi-directional neighbors. Low in most other metrics, particularly thanks rate
**Elite**	Highest in- and out-degree, thanks rate, number of posts, posts per subforum, and typically had posts with low word counts
**Low Volume Supporter**	Typically, moderate in all metrics, however, often a high number of questions per post and long posts
**Information Provider**	Longest posts by a substantial measure with the highest number of URL links and a high initiation rate. Moderate in most other metrics

**Table 4:** Cluster centers for community A. Red to green coloring indicates the lowest to highest values per metric (row).

Input Variable	Overall	Newbie	Popular Supporters	Taciturn	Conversat-ionalist	Elite	Low Volume Supporter	Information Provider
In Degree	40.68	6.26	83.43	8.75	9.22	234.83	14.96	11.58
Out Degree	42.41	3.97	87.38	8.76	8.11	255.16	14.46	10.08
Total Posts	78.52	6.40	133.74	10.00	15.96	610.97	18.12	22.32
Mean Word Count	107.76	157.44	91.42	75.39	107.01	98.90	115.44	279.35
Thank Rate	0.71	0.60	1.01	0.57	0.50	1.13	0.59	0.74
% Questions per Post	0.29	0.77	0.28	0.07	0.22	0.30	0.40	0.22
% URLs per Post	0.07	0.03	0.06	0.03	0.03	0.06	0.05	0.66
Mean Posts Per Thread	2.06	1.91	2.53	1.34	4.78	2.65	1.86	1.78
Initiation Ratio	0.21	0.55	0.09	0.16	0.56	0.08	0.17	0.37
Mean Posts Per Subforum	8.56	2.94	16.25	2.51	8.00	37.77	4.31	4.72
% Bi- directional Neighbors	0.21	0.12	0.32	0.09	0.59	0.45	0.18	0.18

**Table 5:** Cluster descriptions for community B. See Table 6 for more detailed information on cluster centers.

Cluster/Role Name	Description
**Elite**	Highest in- and out-degree, thanks rate, number of posts, and posts per subforum. Typically, their posts had low word counts.
**Newbie**	Highest overall number of questions asked in the community, and typically long word counts in their posts. Low in all other metrics.
**Low Volume Supporter**	Moderately low in all metrics, however, a high initiation rate.
**Popular Supporter**	High overall metrics, particularly mean posts per subforum, in-, out-, and bi-directional neighbor degrees. Low initiation ratio and URLs in posts. Similar to Elite, however, overall lower in each metric.
**Conversationalist**	Highest initiation rate, high word counts, and bi-directional neighbors. Low in- and out-degrees, and thanks rate.
**Taciturn**	Lowest in all metrics, aside from slightly higher connectivity (in- and out-degree).
**Information Provider**	Longest posts, highest number of URLs per post and initiation rate. Lowest in all other metrics.

**Table 6:** Cluster centers for community B. Red to green coloring indicates the lowest to highest values per metric (row).

Cluster Input	ALL	Elite	Newbie	Low Volume Supporter	Popular Supporter	Conversat-ionalist	Taciturn	Information Provider
In Degree	28.66	149.05	8.24	12.96	45.37	5.38	8.61	1.95
Out Degree	29.44	158.89	8.32	10.97	46.90	3.65	9.16	0.87
Total Posts	72.24	489.44	12.75	22.74	92.15	13.64	11.74	5.23
Mean Word Count	118.04	96.00	150.16	118.02	116.49	163.88	67.08	217.03
Thank Rate	0.62	0.89	0.66	0.61	0.64	0.50	0.63	0.19
% Questions per Post	0.29	0.30	0.67	0.23	0.30	0.28	0.10	0.28
% URLs per Post	0.08	0.03	0.03	0.08	0.04	0.06	0.03	0.77
Mean Posts Per Thread	2.24	2.50	1.85	1.73	3.79	2.27	1.41	1.25
Initiation Ratio	0.28	0.13	0.12	0.44	0.10	0.95	0.04	0.77
Mean Posts Per Subforum	10.06	39.90	3.90	5.87	15.44	6.66	3.46	3.60
% Bi-directional Neighbors	0.26	0.51	0.19	0.19	0.42	0.31	0.12	0.09

### Mapping clusters to the reader-to-Leader Framework

Each role will belong to the leader, collaborator, or contributor categories from the RtLF. Readers were not directly included, as they were originally described as users who are “*venturing in*, *reading*, *browsing*, *searching*, *returning*”, [25], hence they have no engagement and passive behavior without a digital trace. These users may not have created an account until they became a contributor, collaborator, or leader, as both forums at the time of data collection were open to view and browse.

We assessed the similarity of clusters identified for each community. While, there are subtle variances in each role identified, we found that they are similar enough to map to the RtLF in the same way. For example, the Popular Supporters in community A and B were subtly different. Community A’s Popular Supporters had high thanks rates, whereas this was not as reflected in community B. However, both community A and B’s Popular Supporters had high in- and out- degrees, bi-directional neighbors, with high mean posts per thread and subforums. Both community’s Taciturns were low in all metrics, however, community B’s tended to be more connected (e.g., higher in- and out-degree scores). Similarly, both community’s Elite users had much in common (e.g., high in- and out- degrees, bi-directional neighbors, thanks rates, and number of posts). However, community B’s number of URLs was a much lower value for Elite users. This highlights subtle differences between forums. This is to be expected, in light of social role theory and literature that addresses the dynamic between the individual and group identity, and shows that there is negotiation and changes in user behavior and adoption of (new) beliefs as individuals integrate into groups and as communities evolve [31]. Further, this aligns with the work of Chan, Hayes, and Daly [32] where they demonstrated the unique role compositions of different forums.

**Fig 3:**
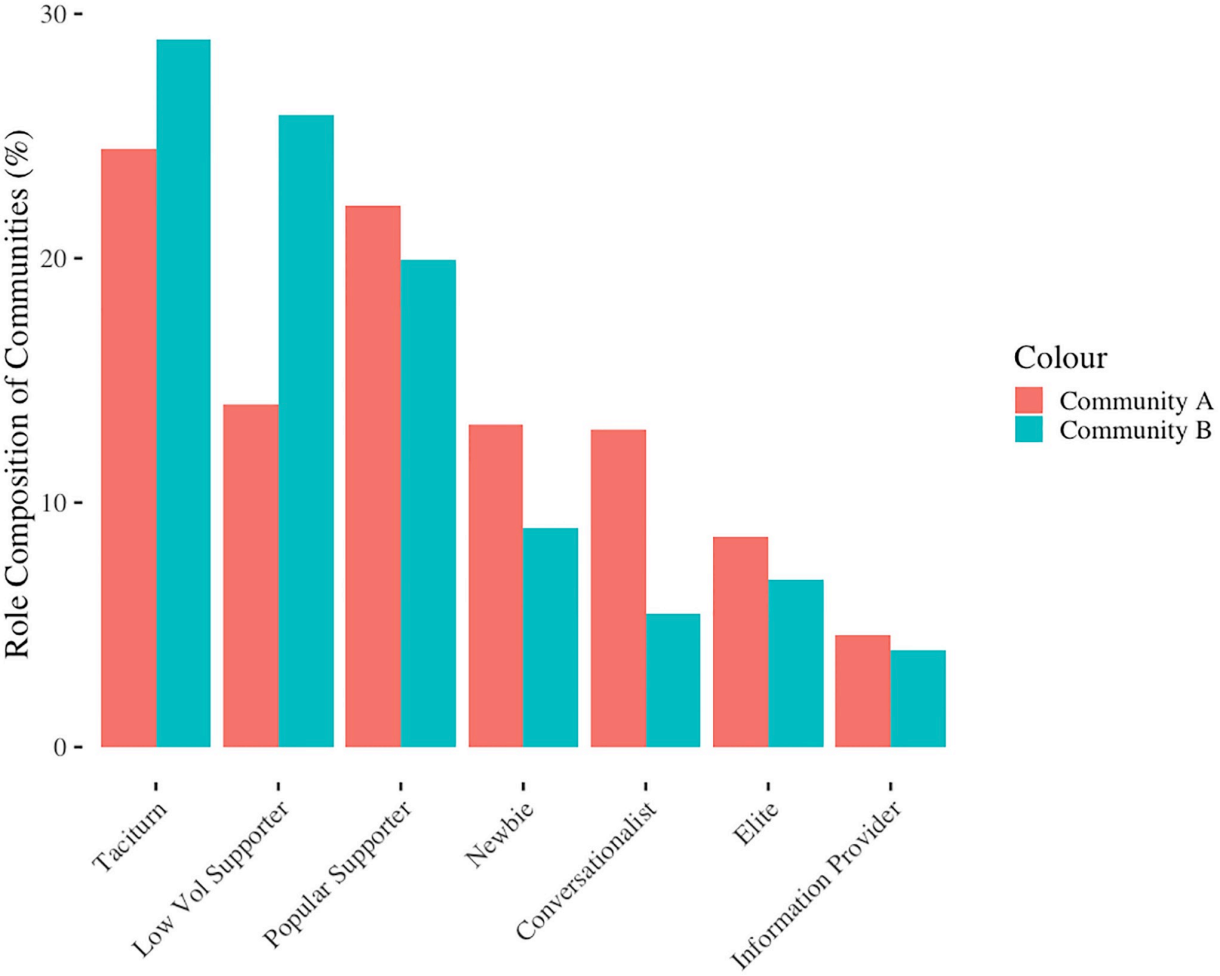
Proportion of users in each cluster for community A and community B.

Next, we examined the proportion of users in each of the roles for both community A and community B (Fig 3), for comparison against the RtLF, as well Nielsen’s 90-9-1 rule of social media and online community engagement [26]. We anticipated seeing larger numbers of users in roles such as Low Volume Supporter or Taciturn, in comparison to Elite users, which was accurate for both forums.

Communities A and B were similar in terms of numbers of users in each role. For instance, in both platforms the highest number of users fell into the roles of Low Volume Popular Supporters, Taciturns, and Newbies. Interestingly, community A had a higher proportion of users falling into the Conversationalist role than community B, and community B had a higher proportion of Low Volume Supporting users. This further demonstrates the subtle differences in role composition in online communities [31,32]. Preece and Schneiderman [25] suggest that reputation is associated with roles, where leaders would have the highest reputation, followed by collaborators with a moderate reputation, and contributors with lower reputation. Hence, the collected reputation scores (inbuilt metrics within the community forum software) were used to further inform this stage. The reputation score for each user is calculated as a function of their total number of posts and reputational upvotes or downvotes from other community members (which are weighted by the reputation *power* that other users wield). However, we acknowledge that the precise details for how the reputation metric is calculated is unknown, which is a key limitation. Hence, we place higher importance on the key features of each cluster and the number of users within each cluster, then we consider reputation as an additional guideline. Reputation was often helpful with our conceptualized metrics, for example, in both forums the reputation score of Elite users far exceeded that of all other clusters.

We noted that the Information Providers and Popular Supporters in community B had lower reputation scores than expected. With consideration of their key features (e.g., in- and out-degree, thanks rates, longer posts), we decided that the Information Providers and Popular Supporters fulfilled the criteria to be a “collaborator” (“*developing relationships*, *working together*, *setting goals*” [25]).

**Fig 4:**
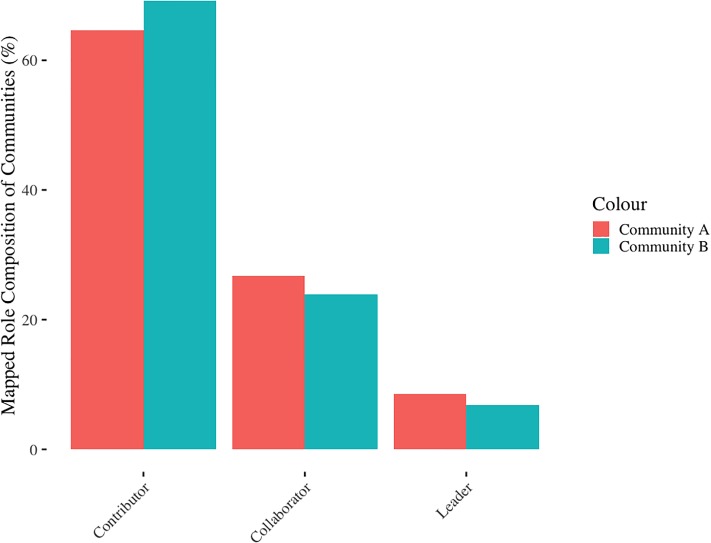
Proportion of users in each category of the reader-to-Leader Framework (RtLF) for A (A) and B (B).

Fig 4 shows the proportion of users belonging to clusters mapped to either contributor, collaborator, or leader, for communities A and B (Fig 4).

Both forums had high numbers of contributors, moderate number of collaborators, and few leaders, which aligns with the RtLF. Despite differences in the proportion of users in the unmapped roles (Fig 3), when mapped to the RtFL, these differences are reduced. Community A’s distribution of users as contributors, collaborators, and leaders was 69.22%, 23.91%, and 6.87%, respectively, whereas community B’s users were 64.66% contributors, 26.74% collaborators, and 8.60% leaders.

### Discussion

Study One aimed to first identify roles using behavioral meta-data. Second, we analyzed these roles by framing them in terms of the RtLF [25]. From our scraped meta-data, we found seven clusters in communities A and B. They ranged from low engagement and passive users, such as Taciturns, to Information Providers, with high levels of engagement, to Elite users, with the highest levels of popularity and thanks rates across the community.

This study shows that various types of role and levels of engagement can be detected within online communities via cluster analysis. These roles can be used to better understand the social structure within online communities. Preece and Schneiderman [25] state their framework is not exhaustive, however, it is dynamic and captures the majority of user behavior online. The contribution of this research is a deeper exploration of contributors, collaborators, and leaders, alongside the identification of “sub-roles” that sit within each category from the RtLF. Further, seeing user types at a higher resolution than the RtLF provides deeper insight into the subtle dynamics within a community. For instance, there were differences in frequency of each individual role between communities A and B, however, these were less noticeable and could be missed if we had only considered three roles: contributors, collaborators, and leaders. We note there were subtle differences in specific role categories, which is to be expected across online communities. Our findings align with Chan, Hayes, and Daly [32], showing that forums are unique communities with different compositions of roles. A further explanation for these variations could be the size differences of the forums, as B (N = 849) is approximately half the size of A (N = 1631). However, a key limitation of this study is that we were limited to meta-data only. It may be possible to shed further light on the subtle differences between online communities by also analyzing the linguistic content. Further, we acknowledge methodological limitations, such as the use of K-MEANS clustering algorithm. Due to the dependence between several metrics, we utilized K-MEANS as no assumptions would be violated, specifically the lack of independence in our variables. The trade-off is the potential sensitivity of K-MEANS, although we mitigated this by utilizing several methods to find a suitable number of clusters, *k*, and thoroughly checking cluster centers against theory and literature. We believe this was the most suitable clustering algorithm for this study due to its ability to handle high-dimension datasets and its increasing use in behavioral analytics [6,27,32,46,47].

Study One shows that we can detect various types of users from behavioral meta-data alone. We build on this in Study two, where we analyze user behavior from community A over time–whether these roles tend to be stable for the user, if they tend to change, and whether the number of users in each role stays consistent. This has several implications for community managers who moderate online communities, marketers looking to identify potential influencers and endorsers of brands, and there may also be implications for security contexts.

## Study Two: Role transitions & community health

Study Two focused exclusively on community A. First, we classified users into the roles from Study One for each of the additional three six-month time slices. This allowed us to analyze the stability of users’ roles and calculate the most common role transitions that users experienced. This aimed to investigate if users shift over time, and more specifically, if (and how) users became leaders. Here, we are most interested in understanding whether users do indeed change behavior as they continue their engagement with this community. Further, this analysis also revealed community A’s high user churn, despite the overall increase in number of users. In addition to the six-month time slice in study one, we used three additional six-month time slices for the period up to the 24 months before data collection date (Table 7). The time slice used in Study One was used as a training set for a Naïve Bayes classifier utilized in Study Two.

**Table 7:** Each six-month time slice and number of users in community A.

Time Period	Number of Users
-24 months to -18 months	1293
-18 months to -12 months	1458
-12 months to -6 months	1495
-6 months to time of data collection	1631

### Classification of community A’s users over time

During the classification step, we used a Naïve-Bayes algorithm, which is a type of generative classifier [53]. This works by taking the inputs (here, these were the metrics found in Table 2) and making predictions of the label (here, this would correspond to the clusters revealed in Study One), where the user is assigned to the most likely cluster they belong to [53]. We classified the final three time slices to the roles detected in the Study One, which allowed us to examine the stability of the roles (mapped and unmapped to the RtLF) over time.

Upon classification, we performed various sensitivity analyses, shown in Tables 8 and 9 below. We note that the sensitivity and accuracy measures show reasonable classification performance. Particularly, as seen in Table 8, the ROC Area for all clusters was, on average, 0.96, which is regarded as “excellent” [54]. This is further demonstrated by the high true positive (TP) rate (column 1) and the false positive (FP) rate remaining low (column 2).

**Table 8:** Classification accuracy by cluster (%).

	TP Rate	FP Rate	Precision	Recall	F Measure	MCC	ROC Area	PRC
**Newbie**	0.87	0.06	0.58	0.87	0.69	0.68	0.95	0.87
**Popular Supporter**	0.87	0.03	0.88	0.87	0.88	0.85	0.98	0.93
**Taciturn**	0.88	0.05	0.88	0.88	0.88	0.83	0.98	0.96
**Conversationalist**	0.60	0.01	0.79	0.60	0.68	0.67	0.97	0.73
**Elite**	0.99	0.01	0.89	0.99	0.94	0.93	1.00	0.96
**Low Volume Supporters**	0.62	0.07	0.76	0.62	0.68	0.59	0.92	0.78
**Information Provider**	0.92	0.01	0.77	0.92	0.84	0.84	0.96	0.87
**Weighted Avg.**	0.80	0.05	0.81	0.80	0.80	0.76	0.96	0.88

**Table 9:** Confusion matrix for classification (%). Actual values as rows; predicted values as columns.

	**Predicted**							
**Actual**		**Newbie**	**Pop Sup**	**Taciturn**	**Conver.**	**Elite**	**Low. Vol. Sup.**	**Info. Prov.**
	**Newbie**	**7.79**	0	0	0.25	0	0.67	0.25
	**Popular Supporter**	0	**17.41**	0.12	0.12	0.8	1.35	0.12
	**Taciturn**	0.18	0.61	**25.38**	0	0	2.51	0.25
	**Conversationalist**	0.55	0.25	0.49	**3.25**	0	0.61	0.31
	**Elite**	0	0.06	0	0	**6.81**	0	0
	**Low Volume Supporter**	4.97	1.41	2.82	0.43	0	**16.06**	0.18
	**Information Providers**	0	0.06	0.06	0.06	0.06	0.06	**3.68**

Table 9 is the confusion matrix from our classification step, which provides more detail regarding which clusters were less accurately classified. The highest area of sensitivity in the classification model often concerned Low Volume Supporters. They were slightly more likely to be misclassified due to the lack of distinctive features (e.g. they lacked particularly high or low metrics for certain behaviors), unlike the other roles. There is also potential for greater sensitivity among the lower contributing roles, as each of their interactions may have more impact on the overall metrics. However, we wanted to keep the low engagement users in our analysis (e.g., low volume supporters) in order to reflect the broad spectrum from non-engagement to high engagement users presented in the RtLF. This also allowed us to capture users who may be more likely to join and leave swiftly, as shown in our analysis of churn within the community. Hence, if we removed these users, we would lose subtle sub-roles that are an important section of the community user base. We reiterate that we are unable to capture readers, since they do not leave a digital trace.

**Fig 5:**
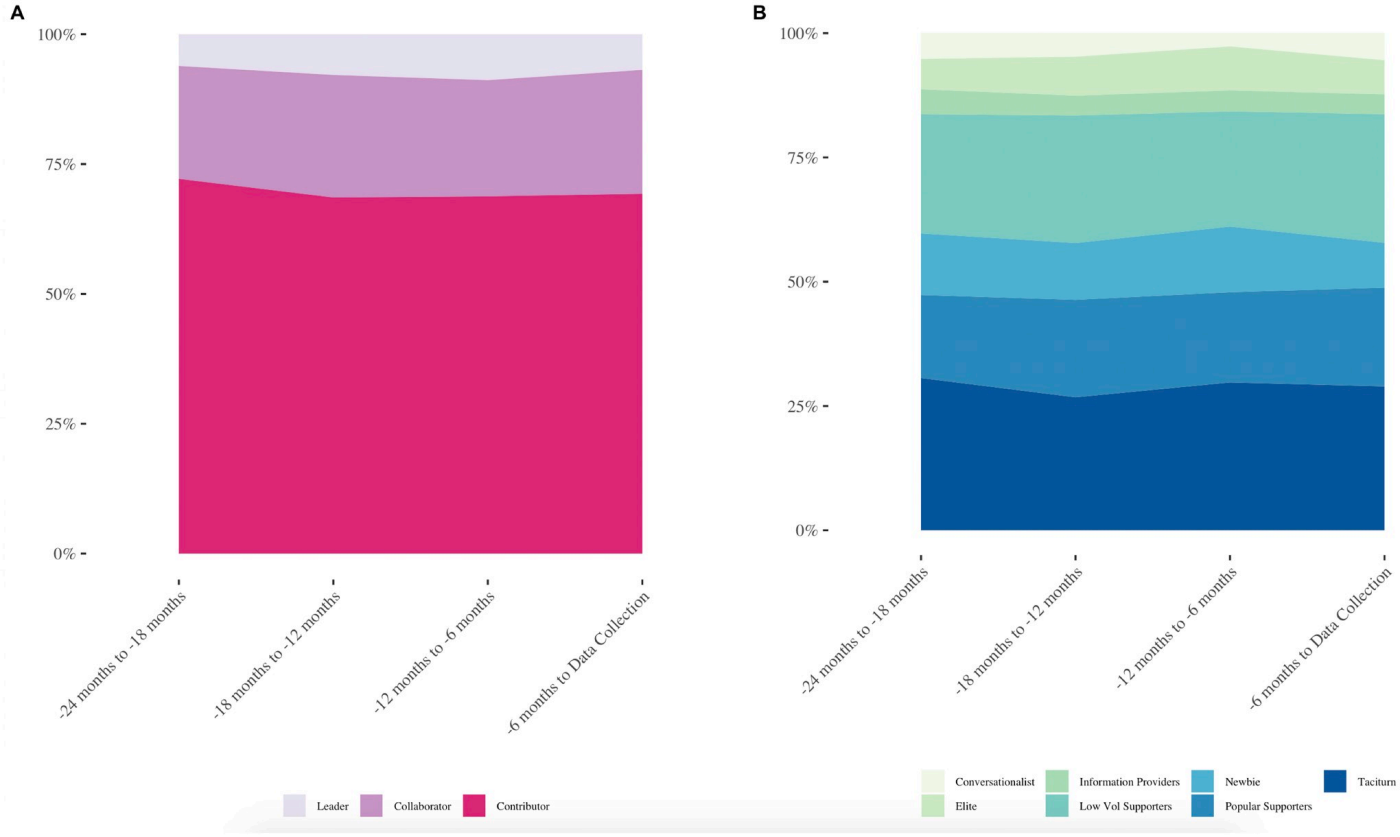
Percentage of users in each role—Mapped to the RtLF (A) and unmapped to the RtLF (B).

Fig 5 shows that the percentage of users in each role is moderately stable and consistent. We can see this is particularly prominent with the Elite and Information Providers, as well as contributor roles like Low Volume Supporters and Taciturns. We noticed the increase in Low Volume Supporters in the most recent time slice. There are relatively subtle fluctuations in overall RtFL categories. However, in the most recent time slices the numbers of users in each mapped role (contributors, collaborators, and leaders) were remarkably stable, despite the amount of churn in members leaving and joining the community. We found 25.32% of users from the earliest time slice were present at the time of data collection 24 months later. While substantial numbers of the community left at each time slice, the overall size of the community grew over the two-year time period, which reveals that large numbers of new users also joined.

### User role pathways

Next, we examined user role changes over the two-year period. We analyzed every possible cluster transition that users could make (N = 64, i.e. switching between the 7 clusters, and also transitions to an inactive (reader) state). 7,712 user transitions were observed (i.e., comparisons of an individual’s role from one time slice to the next), which included users that remained in the same cluster, changed once, or even multiple times. Users could change up to three times (across the four time slices). Transitions were identified simply by comparing individual user’s cluster allocations across consecutive time slices. Only users who had appeared in earlier time slices were included in any subsequent inactive to inactive transitions (i.e., users who had not yet joined the community are not counted).

Table 10 shows the *top ten most common transitions* seen within community A across all time slices. The frequency of each transition pathway was counted, which formed the basis for calculating the most common pathways.

**Table 10:** Top Ten most common pathways for users to take based on all possible transitions (N = 7,712).

Pathway (Clusters)	Number of Users	% of Users
Inactive → Inactive	1082	14.03
Inactive → Taciturn	834	10.81
Taciturn → Inactive	780	10.11
Low Volume Supporter → Inactive	629	8.16
Inactive → Low Volume Supporter	480	6.22
Newbie → Inactive	367	4.76
Inactive → Newbie	335	4.34
Popular Supporter → Popular Supporter	324	4.20
Inactive → Popular Supporter	292	3.79
Low Volume Supporter → Low Volume Supporter	195	2.53

Four out of the top ten pathways in Table 10 regard users becoming inactive or remaining inactive. Hence, it was common for users to disappear from the community (or assume a reader role) from one time slice to the next. Four out of the top ten concerned new joiners or those becoming active again, where three of these pathways were users becoming contributors, and the other pathway reflecting users going straight to a collaborator role. The final pathway were users who remained contributors or collaborators.

**Fig 6:**
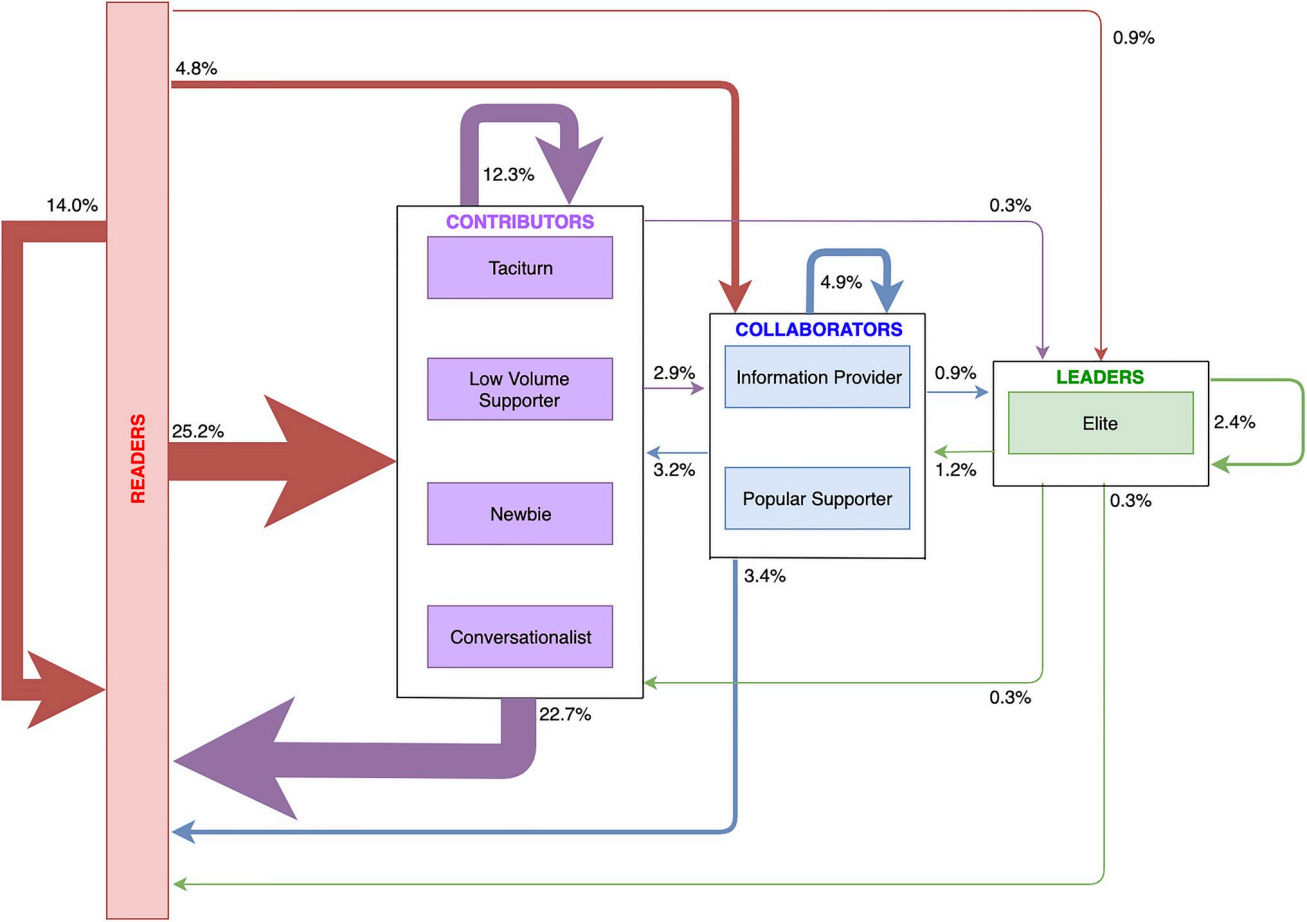
Model showing percentage of all users role transitions mapped to the reader-to-Leader Framework.

We developed a model from the all user transitions of community A (Fig 6). This model mimics the RtLF and highlights the diminishing numbers of users progressing to leadership within the community. From Table 10, if we consider the percentage of *all transitions* users made (N = 7,712), only 2.9% of those transitions were of users shifting from a contributor to a collaborator, and only 0.9% of transitions were collaborators transitioning to leaders. Few transitions concerned users joining as leaders (0.9%), however, it was slightly more common to join as collaborators (4.8% of transitions) or simply a contributor (25.3% of transitions).

Fig 6 provides a high-level overview of how users changed their roles over time. This considers *all transitions made* in in the dataset. It reveals that users staying in the same RtLF category is common, for instance, we found 12.3% of transitions were users that were once contributors and remain contributors, similarly, 4.9% of transitions were users that did not change from a collaborating role, and 2.4% transitions were users that remained leaders. Fig 6 also demonstrates the high churn of users, where 30.9% of transitions regarded new joiners or those returning from a period of inactivity, whereas 26.4% of the transitions were users changing back to readership/inactivity. This also demonstrates that the community within the two-year time period of data grew overall.

**Fig 7:**
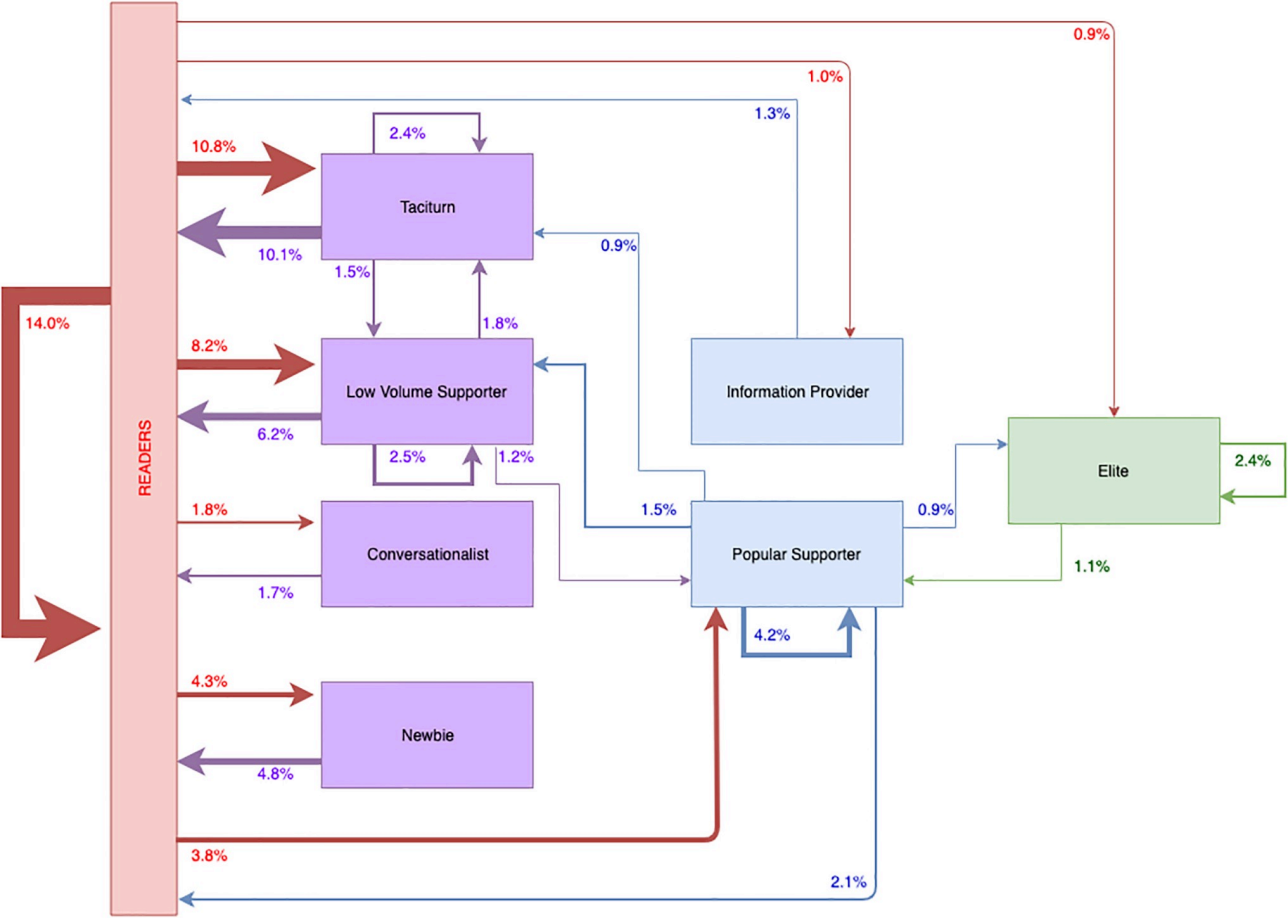
Model showing the top 25 transitions of users role transitions. Demonstrating the linear and non-linear pathways users took.

In addition to Fig 6, we present Fig 7, which shows the top 25 transitions users made. This, in contrast to Fig 6, is not mapped against the RtLF, which enables us to see the intricate (role level) pathways users took. It also shows the most common pathway users took to become a leader (although this appears to be exceptionally rare in community A). It further demonstrates non-linear pathways of users (e.g., demoting transitions–Popular Supporter to Taciturn).

From Fig 6, we saw that 25.2% of transitions during the two-year period, transitioned from readership to a contributor. Fig 7 provides a higher resolution of this and shows that the majority of transitions from readers into contributor roles were users becoming Taciturns, followed by Low Volume Supports, then Newbies, and much less commonly, Conversationalists. We also found a fairly common cyclical role pathway of users either remaining Taciturns or Low Volume Supporters or switching between the two. Therefore, users are more likely to switch between these roles or become inactive rather than progress towards leadership.

If we consider the paths of progression towards leadership, out of all transitions made, it was exceptionally uncommon for any contributor (Taciturn, Low Volume Supporter, Conversationalist, or Newbie) to become an Information Provider. Instead, it was more common for a small number of readers to jump straight to this role. Within the *top 25 most common transitions*, the only contributor role that led to a collaborator role was Low Volume Supporters transitioning into Popular Supporters. However, it was actually more common for Popular Supporters to be demoted back to Low Volume Supporters. Out of all transitions made, 0.9% of transitions progressed from Popular Supporters to the leadership role of the Elite. In terms of the top 25 most common transitions, the most common path to the Elite is: **Reader → Low Volume Supporter → Popular Supporter → Elite**. While, there were other pathways identified, these were exceptionally rare routes that users followed in comparison.

## General discussion

We presented two studies that reveal insightful information about the composition and dynamics of an online community based on meta-data alone. For instance, uncovering specific behaviors associated with particular subsets of users within a community, known as roles. These roles can be framed within the RtLF, which helps to reveal which users are in leadership positions and which are less engaged. The first study analyzed the meta-data from two ideological communities (A and B). We found seven clusters via K-MEANS cluster analysis, which we analyzed and formulated into different roles within each forum. These roles were mapped against the RtLF, based on the key features of each role, the number of users in each role, and the average reputation score of each role. The findings from both studies have potential to be used to predict user role changes in future.

Further, the method is a key contribution, as it provides a way to reveal and further examine groups of users over time, providing insight into user dynamics within various online communities. This information is useful for a variety of contexts, for instance, targeting marketing campaigns for specific groups of users and across different contexts or identifying potential new influencers for brands. In addition, this could be of interest to security practitioners as this work, and our methods, may provide insight into which users may be leading and guiding narratives, and a way to identify potential users of interest.

### User role compositions

When comparing the two forums, we found similar roles, however, there were some specific differences. For instance, the Popular Supporters in community A and B were subtly different. Where community A’s Popular Supporters had high thanks rates, this was not as reflected in community B. However, they were similar in all other metrics (e.g., in- and out-degree, bi-directional neighbors). As noted in Fig 3, there are differences in the proportion of users in each role for community A and B, where community B had a higher proportion of Low Volume Supporters, however, community A had a higher proportion of Conversationalists. These differences were to be expected, as each online community is its own eco-system consisting of different roles, proportions of roles, and individuals within it [32]. Further, the system itself can impact the way in which users behave, thus influencing the expressed behaviors of each role [55].

Once we had described and developed the roles based on features, frequency, and reputation scores, we then mapped these roles to the RtLF. Both forums had high numbers of contributors, many collaborators, and few leaders. However, we were unable to capture “readers”, who are “*venturing in*, *reading*, *browsing*, *searching*, *returning*”, [25], as they would not have generated any behavioral meta-data captured via scraping. We analyzed these roles, both mapped (to the RtLF) and unmapped, in community A over a two-year time period (Study Two). This was to firstly provide insight into how stable these roles are over time and what this implies about the health of an online community. Secondly, we considered how users changed over time and which role transitions were most common. Further, this allowed us to examine user churn, which provided further insight into the intricate social dynamic of the community.

Interestingly, we found there was a large churn of users at each time slice [11,12]. However, as seen in Table 7, the overall size of community A grew at each time slice. Despite this large turnover of users over time, and the constant influx of new users or returning users from a period of inactivity, as seen in Fig 5, the proportion of users falling into each role (mapped or unmapped to the RtLF) was remarkably stable. This has implications for the overall health of an online community [22], which will be discussed further in the following sections. Focusing specifically on the user role compositions, both mapped and unmapped, based on Figs 3 to 5, we see a calm and stable appearance of user turnover and proportions of users in each role. However, there is a flurry of role transitions taking place beneath the surface.

### The pathway to leadership

We revealed users changing roles across each time slice–rarely moving in a linear fashion through the RtLF, but more commonly, staying in the same RtLF category, or demoting their role. We reiterate that Figs 6 and 7 and Table 10 are based on the number of users who made each transition over *all time slices*.

We analyzed every transition each user made (N = 7,712) and discovered that the majority of transitions were users becoming inactive or users who remained inactive (40.4%). This aligns with literature looking at user participation, motivation, and the retention of users [2,24,25]. It is often difficult to retain users and motivate them to engage [23], which was seen within community A, where the churn of users was high. However, we discovered that the second most common set of transitions were new users joining the community into contributor and collaborator positions, which aligns with our findings that despite the high churn of users, the overall community grew in size over the two-year time period. Other relatively common pathways include a cycle between Taciturns and Low Volume Supporters, which consists of users who showed extremely low engagement as Taciturns and somewhat higher levels of engagement as Low Volume Supporters (Table 4). Typically, users in these roles (as contributors more generally) either remained in a contributing role (Fig 6) or became inactive, as shown in Fig 7. These non-linear movements are to be expected with online communities, as reflected in the RtLF [25] and in Nielsen’s Rule of Internet engagement [26] with the vast majority of users engaging and contributing little.

Perhaps one of our most important empirical findings relates to the rarity of users becoming leaders within community A. We demonstrate that astonishingly few users did progress linearly through the RtLF, and following this pathway to the Elite role is against the odds. However, the exceptional users who were leaders, often stayed leaders. In contrast, contributors were far more transient in nature, shown by increased numbers transitioning to inactivity from those roles. However, based on Fig 7, we did discover the most common pathway users would take to become a leader:

**Reader → Low Volume Supporter → Popular Supporter → Leader**

Out of all transitions that users made (N = 7,712) in the two-year period, only 1.2% of transitions were from Low Volume Supporters to Popular Supporters, and 0.9% of all transitions involved progression to Elite from Popular Supporter roles. There were other pathways users could take to become leaders, however, they were extremely rare (and were subsequently not captured in Fig 7). This is perhaps not surprising, as other work has shown that recruitment and mentoring of new editors in online communities such as Wikipedia remains a challenge [56]. Hence, we suggest there may be a similar lack of mentorship from Elite users, which contributes to the few users becoming leaders in community A. However, this would need to be investigated further using content data.

### User roles and community health

Within community A, we found that roles, both mapped and unmapped to the RtLF, remained consistent over time. It may be that a healthy online community has a level of stability and consistency of role distributions over time, aligning with Angeletou et al., [22]. If we consider consistent roles alone as a sign of health within a community, we would argue that community A is an example of a healthy community (it only closed recently due to financial reasons—several years after the data was collected for the present research). Angeletou et al. [22] also noted that increasing levels of “ignored” users will decrease the overall health of a community. Examples of “ignored” users in our analysis were Taciturns, due to their low engagement and overall contribution. We found the numbers of Taciturns remained stable over time, further indicating a healthy community. We also align with Soroka and Rafaeli [24], where they propose lurking behavior is unlikely to be harmful, as these users may not have content to contribute. They argue that enabling readership without contribution is helpful to maintaining a healthy community, as it can reduce noise and clutter across forums. If we consider that elite users are there to guide and influence the community, too many users attempting to do this could lead to detrimental effects on the community.

We must recognize that there are differences for what constitutes “healthy”. For instance, the specific nature of a community, as types of roles, and the composition of those roles within the community will naturally vary [32]. This provides a potential avenue for further research. In the present study, we have focused on ideological communities. We anticipate there could be differences in non-ideological communities due to the nature of the content shared or the purpose of use as demonstrated by Chan, Hayes, and Daly [32].

## Limitations

First, referring back to Table 2 and the metrics employed, it is important to understand how these relate to behavior presented by users. While these metrics are ego-centric, some features (e.g., structural and reciprocity) also rely on the other community members for feedback (e.g., thanks rate). Features like this are critically important for understanding leading users, as we would anticipate these users to be popular and well-regarded. However, we must note that each community has its own eco-system of roles, which will naturally have different behavioral features [32,57], therefore, the optimal metrics to identify particular subsets of users with particular behavioral patterns might differ from community to community. The key limitation here is that, despite the metrics developed for this work, meta-data can only provide a certain level of information. Analyzing the forum content data or performing a network analysis, for example, would likely reveal further insight about the intricacies of specific online communities.

Second, we acknowledge that the overall category of “contributor” is wide in our dataset, where we have included users with an extremely low levels of engagement (e.g., <10 posts). This can cause difficulty with classification, as noted in Table 9, where Low Volume Supporters were the most likely to be misclassified. However, as stated previously, these users were kept in the dataset as we aimed to capture the entire community, especially those closer to the uncaptured “readers”. This is an important section of the community to include, as these may be users that have just joined, or are close to leaving the community. We also note that extremely low activity users could create highly sensitive metric values, which may have an impact on the accuracy of the clustering values. However, as seen in Tables 8 and 9, where misclassification was low, we do not see strong evidence that this was a significant issue.

Finally, the time slices used (six months) are also wide. We selected this window primarily as we wanted to ensure that changes in behavior reflected significant role changes, rather than capturing temporary fluctuations of engagement. We do acknowledge, however, that some users may have changed multiple times within the six-month time period, which were naturally uncaptured here.

## Future work

The present study demonstrates that we can examine user behavior to gain understanding of how their role within an online community may change over time. This provides the basis for future research directions. First, one might replicate this across a variety of online communities. This would offer insight into the differences between different types of online communities. This could also utilize more than just meta-data alone (e.g., content, linguistic features), perhaps employing qualitative or ethnographic approaches to reveal other subtleties.

Second, there is work to be done regarding metric development from metadata (and other data types). Our metrics have been based on literature [32], as well as adding some additional features. We note that due to low activity users being of interest, there are potentially better ways to handle measurements on various engagement metrics such that the overall sensitivity is reduced. The meta-data we used was derived from public postings, but many other forms of data would be available to forum administrators (e.g. profile updating, post deletion, length and frequency of access) that would be useful in building models of user behavior.

Third, further work may consider the use of different theories to ground the modeling, for example, the use of social identity theory to consider in- and out-group differences between roles or communities. Although, this again, would benefit from utilizing more than just meta-data alone.

Fourth, we acknowledge the time slices we used in this work (six months), are fairly wide. Further work could explore different sizes of time slice and what different time slices could offer in terms of understanding user behavior (e.g., subtle versus substantial changes in behavior, temporary or sustained).

Finally, and perhaps most importantly, our empirical findings have highlighted an area of future work relating to understanding moderation, mentoring, and other potential mechanisms that could be used to foster and develop new users [56]. This could reveal ways in which we can create the new leaders of online communities by making rare pathways to leadership more widely known and accessible for new joiners.

## Conclusions

Online communities have the power for good–to support those in need, to create a shared collective consciousness, and to exchange information and ideas. However, this powerful influence can also be utilized conversely. For example, there has been a recent up rise with the “involuntary celibate” or “incel” movement [7,8]. We also face a constant battle with radicalization online [58], which remains difficult to understand and intervene. We have presented a novel method to examine roles within an online community, which has utilized the Reader-to-Leader Framework [25] in order to help conceptualize types of users in terms of leadership. This approach has the potential to be highly valuable in contexts where the role evolution of online forum users needs to be investigated. The demonstrated method can be applied to a variety of online behavioral meta-data and used by researchers and practitioners interested in understanding online communities from a data-driven perspective. This research also has implications for community managers and moderators wishing to assess the health of community by understanding role distributions and dynamics, and security analysts wanting to identify how leadership positions are occupied in malevolent online communities.
